# Hard-Baked Photoresist as a Sacrificial Layer for Sub-180 °C Surface Micromachining Processes

**DOI:** 10.3390/mi9050231

**Published:** 2018-05-11

**Authors:** Hani H. Tawfik, Mohannad Y. Elsayed, Frederic Nabki, Mourad N. El-Gamal

**Affiliations:** 1 Department of Electrical and Computer Engineering, McGill University, Montreal, QC H3A 0E9, Canada; mourad.el-gamal@mcgill.ca; 2 MEMS Vision Intl., Montreal, QC H1Z 2K4, Canada; mohannad.elsayed@mems-vision.com; 3 École de Technologie Supérieure (ETS), Montreal, QC H3C 1K3, Canada; frederic.nabki@etsmtl.ca

**Keywords:** Silicon carbide (SiC), surface micromachining, photoresist, sacrificial layer, low thermal budget

## Abstract

This letter proposes a method for utilizing a positive photoresist, Shipley 1805, as a sacrificial layer for sub-180 °C fabrication process flows. In the proposed process, the sacrificial layer is etched at the end to release the structures using a relatively fast wet-etching technique employing resist remover and a critical point dryer (CPD). This technique allows high etching selectivity over a large number of materials, including silicon-based structural materials such as silicon-carbide, metals such as titanium and aluminum, and cured polymers. This selectivity, as well as the low processing thermal budget, introduces more flexibility in material selection for monolithic integration above complementary metal oxide semiconductor (CMOS) as well as flexible substrates.

## 1. Introduction

Micro-electro-mechanical systems (MEMS) have helped in the miniaturization of various sensors and actuators that include, but are not limited to, resonators, inertial sensors, ultrasonic transducers, and RF switches [1]. MEMS sensors or actuators are either micromachined on top or as part of silicon substrates which are typically referred to as surface (e.g., [2]) and bulk micromachining (e.g., [3]), respectively. The former allows a monolithic implementation of MEMS sensors and actuators on top of complementary metal oxide semiconductor (CMOS) substrates in which the read-out and driving integrated circuits (ICs) are implemented [4]. This is desired for saving system footprint in a large number of applications, such as portable devices. Moreover, surface micromachining presents a more economical choice in some applications such as microfluidic systems, owing to the fact that bulk micromachining implementations require an extra wafer-bonding step for encapsulation [5]. Monolithic integration is crucial for some applications that require a large amount of interconnects between the MEMS and ICs. For example, one can consider thousands of infrared sensors that are tiled to form a 2D focal-plane array to build a thermal imaging camera [6]. Using conventional wire-bonding, this application would require an unrealistic number of connections making IC-compatible surface-micromachined monolithic integration a preferred solution for these applications.

IC compatibility, notably CMOS IC fabrication processes, is a major interest for monolithic integration of MEMS. It necessitates the use of CMOS compatible materials to avoid any contamination of the CMOS substrates [7]. In addition, it requires the limitation of the process to a maximum processing temperature (thermal budget) of 400 °C to avoid the destruction of the lower layers in the CMOS substrate (e.g., interconnects). An additional reduction in the thermal budget beyond the CMOS compatibility limits, i.e., below 250 °C, is needed for integration on top of flexible substrates [8]. Despite the many favored characteristics of surface micromachining for high volume MEMS applications, bulk micromachining has been well-suited in devices requiring large vibrating masses such as inertial sensors [3]. Furthermore, forming high-quality mechanical layers at a low temperature in surface micromachining often requires non-traditional procedures such as laser treatment [8]. Silicon carbide (SiC), in addition to having been introduced as a CMOS compatible material [9,10,11], features excellent mechanical properties such as higher elastic modulus and yield strength values relative to silicon that makes it an attractive choice for MEMS applications. SiC is known as well for its mechanical hardness and electrical stability at temperatures above 600 °C that ensure the reliability and robustness of the fabricated structures [9]. Moreover, it can be deposited at room temperature using sputtering [10]. Recently, the authors in [11] reported a versatile, CMOS compatible, 3D process that uses two structural layers of sputtered SiC on top of aluminum (Al).

The release step in such processes often proves to be a challenge. In surface micromachining, structures are suspended by growing them on top of sacrificial layers that are removed at later steps in the fabrication to release the structural layer intact, and without damaging any other layers, e.g., the metallic interconnect layers. This requires an etching method that is highly selective to the sacrificial layer and that does not react with the device’s structural layer. This limits the number of etchants, consequently sacrificial layers, that can be used without an adverse effect on the interconnects, the structural layer, or the underlying IC. Cured polymers such as polyimides satisfy both conditions when used as sacrificial layers. However, they are etched using isotropic oxygen plasma etching that ashes them away to release the structure [10,11]. For devices with small transducer gaps (i.e., small sacrificial layer thicknesses) and large areas, long release hours are needed that can increase the fabrication cost significantly. In addition, it precludes the possibility of using polymers in MEMS structures or integrating them above flexible substrates.

In this work, the possibility of using hard-baked photoresist as a sacrificial layer for sub-180 °C surface micromachining processes is proposed and demonstrated. The used process utilizes SiC on top of Al as the suspended structural layer, with titanium (Ti) for metal-routings performed under maximum operating temperature of 180 °C. Such a low thermal budget makes the presented sacrificial layer solution a great candidate for integration above CMOS and flexible substrates. The proposed fabrication process flow is detailed in Section 2. The fabrication results are reported in Section 3. Finally, a thorough discussion on the contribution of this work on possible combinations of sacrificial and structural available for surface micromachining is given in Section 4.

## 2. Process Development

**Figure 1:**
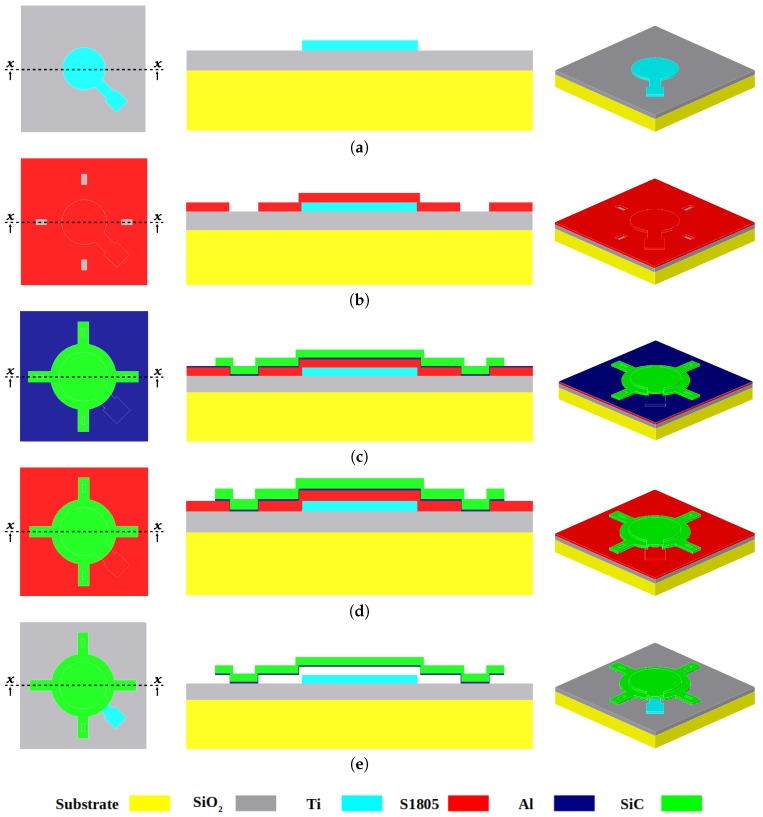
Top-view (left), cross section x−x (middle), and 3D (right) views for the different steps of the proposed fabrication process flow: (**a**) depositing and patterning the lower metal (Ti); (**b**) spinning the sacrificial layer and defining the anchors; (**c**) depositing (SiC + Al) and patterning the SiC structural layer; (**d**) etching the upper metal (Al); (**e**) releasing the structure in ultrasonic bath + critical point dryer (CPD).

A silicon (Si) substrate with 2 μm thermal silicon dioxide (SiO${}_{2}$) layer on top is used. A 500 nm Ti layer is deposited on top of the substrate in a direct current (DC) sputtering equipment and patterned using positive photoresist as shown in Figure 1a. Typically, a sacrificial layer would be deposited and a photoresist layer spin-coated on top of it to be patterned to define the anchors in the sacrificial layer. However, in this work, the first step is skipped, since the patterning layer is the same as the sacrificial layer. Hence, a 500 nm positive Shipley 1805 photoresist is spin-coated on top of the wafer at 4000 rpm for 30 s. This layer is exposed to ultraviolet to pattern the SiC structural layer anchors, as depicted in Figure 1b. The photoresist is then soft-baked at 110 °C for 90 s. After that, it is developed in an MF319 developer for 40 s. Afterwards, a 2-step hard-baking is performed at 130 °C for 3 min and at 170 °C for 30 min on top of a hot plate. The hard-baking is crucial to ensure the survival of the photoresist during the sputtering of the next layers, notably the SiC structural layer. During sputtering, ion-bombardment raises the temperature of the substrate causing the sacrificial layer (i.e., photoresist) to outgas. This results in bubbles that destroy the formed films and causes flakes inside the deposition chamber, hence the need for 2-step hard-baking. A similar approach is reported in [12] where a Shipley 1813 photoresist (used as a sacrificial layer as well) is hard-baked at 175 °C for 3 h in order to protect it during SU8 application.

After that, the stack of the structural layer is deposited using DC sputtering. The stack consists of 2 layers. The first is the upper metal, a 60 nm Al that mimics the function of the top electrode in a capacitive MEMS device, as well as, an etch stop. The second is a 500 nm SiC that forms the main structure. The stack is then patterned using a 1.4 μm Shipley 1813 positive photoresist. To distinguish it from the sacrificial photoresist layer, it is referred to hereafter as the “etch mask”. The SiC is etched using reactive-ion-etching with NF${}_{3}$ for 100 s, stopping on the Al followed by stripping the etch mask in acetone as seen in Figure 1c. It is important to note that the sacrificial layer is not affected by the immersion in acetone at this step since it is hard-baked. However, exposure to NF${}_{3}$ for longer periods of time (to pattern thicker SiC films), toughens the etch mask and it becomes unsolvable in acetone. This only happens to the etch mask photoresist as the sacrificial photoresist is protected by the Al as shown in Figure 1c. Attempts to remove the toughened etch mask resulted in damages in the sacrificial layer. This issue is solved by using chromium as an etch mask.

Subsequently, the Al layer is wet etched in a mix of phosphoric, acetic, and nitric (PAN) etchant for 90 s at 30 °C, thus uncovering the sacrificial layer for the release step, as shown in Figure 1d. After that, the wafer is diced and each die is released, as seen in Figure 1e, in a photoresist remover 1165 solution in a Crest CP1100D (Crest Ultrasonics Corp., Ewing Township, NJ, USA) ultrasonic bath for 45 min. The temperature of the ultrasonic bath is set to 70 °C at half of the maximum ultrasonic power. Finally, the dies are rinsed in an iso-propanol alcohol (IPA) several times then dried in a critical point dryer (CPD) to avoid any stiction problems.

## 3. Fabrication Results

**Figure 2:**
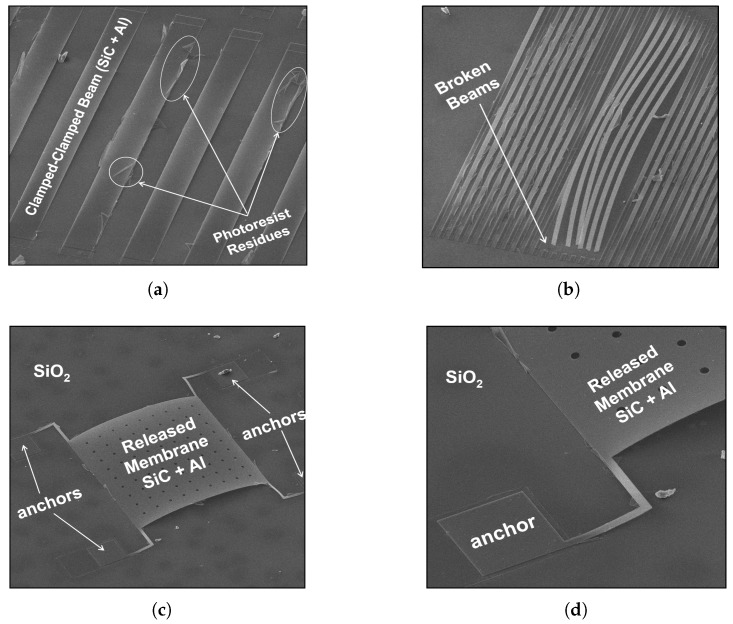
Scanning electron microscope (SEM) images of fabricated and released test structures, using the complete process flow without the Ti layer: (**a**) clamped–clamped beams; (**b**) clamped–clamped beams broken due to high ultrasonic power; (**c**) rectangular membrane suspended by four L-shaped beams; (**d**) close-up of one of the membrane’s anchors.

The proposed fabrication process was performed. In early trials, step (a) shown in Figure 1a was skipped to simplify the process. Hence, only the sacrificial layer and the structural layer stack were implemented followed by the release process (steps (b) to (e) are shown in Figure 1b–e and detailed in Section 2). Scanning electron microscope (SEM) images were taken for the fabricated test structures (Figure 2). Photoresist residues were noticed in early stages. Clamped–clamped beams shown in Figure 2a,b have a significant amount of sacrificial layer residues attached to their edges. Nonetheless, the release process was successfully achieved. This is demonstrated by inspecting structures that suffer high compressive stress. Structural layers with residual compressive stress experience buckling. This bending demonstrates successful release of the fabricated structures as shown in Figure 2c, which depicts a rectangular membrane suspended by four L-shaped beams. A closer look on one of the beams in Figure 2d shows that the L-shaped beam and the membrane are successfully released despite of the sacrificial layer residues.

**Figure 3:**
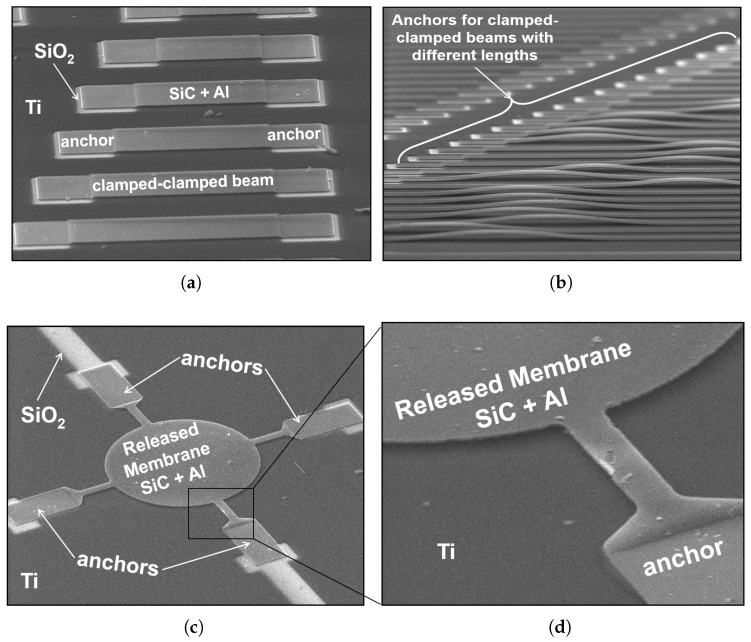
SEM images of fabricated released test structures using the complete process flow: (**a**) top view of clamped–clamped beams; (**b**) side-view of clamped-clamped beams suffering residual compressive stress; (**c**) circular membrane suspended by four beams; (**d**) close-up of one of the beams.

The photoresist (sacrificial) residues problem was solved by renewing the resist remover after 15 min of sonication in the ultrasonic bath as well as increasing its time to 45 min. Maximum ultrasonic power was found to break some of the beams with large aspect ratios as depicted in Figure 2b. This was solved by reducing the ultrasonic power to 50%, yielding good results as shown in Figure 3. The proposed process was performed by including the lower Ti layer at the beginning. All of the Ti, Al, and SiC survived at the end of the fabrication as shown in Figure 3, showing 10 μm-wide clamped–clamped beams with aspect ratios ranging from 1 (Figure 3a) to 100 (Figure 3b). As explained earlier, the latter suffers residual compressive stress that bends the beams upwards, clearly demonstrating a successful release. Contrary to an earlier attempt shown in Figure 2a,b, SEM images for the finalized process show no sign of photoresist (sacrificial) residues as depicted in Figure 3a,b. Similar results were found with different types of structures such as the circular membrane with 85 μm diameter suspended by four beams shown in Figure 3c. A zoomed view of one of the beams is shown in Figure 3d. Shipley 1800 series positive photoresist in the proposed process allows for several thicknesses for the sacrificial layer ranging from 0.5 to 2.5 μm with a resolution down to 0.48 μm. For simplicity in fabrication, the lower metal (Ti) was patterned using the anchors mask as well. The full release of the structures was confirmed as well on the probe station by mechanically pushing the structures using DC needles.

## 4. Discussion

The appropriate choice of the sacrificial layer is of a paramount importance in the design of surface micromachined MEMS devices since it determines the possible structural materials that can be included in the design. Structural layers such as Poly-Si, SiC, and SiN with desirable mechanical properties typically require sacrificial layers deposited at relatively high temperatures (high thermal budget) such as silicon oxide or Poly-Si [5,9,13], hence limiting their suitability for monolithic integration. In addition, their etchants such as tetramethylammonium hydroxide (TMAH) and potassium hydroxide (KOH) for Poly-Si or hydrofluoric acid (HF) would attack metal layers such as Ti and Al etching underlying interconnects. A diluted HF and pad etch could be used to reduce the attack on metal layers. However, long-term exposure to the etchant during the release process degrades the quality of SiN and Poly-Si films [13]. Polymers, such as polyimides cured at low temperatures, are suitable sacrificial layers for monolithic integration. They enable the use of metals for interconnects and reliable mechanical layers such as SiC in the MEMS device [4,10,11]. Nonetheless, the need to release the structures using oxygen plasma rules out the possibility of using a polymer as a structural part of the MEMS device. The use of polymers has been of great interest in various areas in the MEMS world such as microfluidic [14,15] and thermal detectors [15]. Moreover, such sacrificial layers limit the monolithic integration on flexible substrates. Alternatively, the photoresist hard-baked at relatively low temperatures of 115 °C, which can be dissolved easily afterward in acetone, has been the typical sacrificial layer of choice for such flexible substrate applications. Releasing the MEMS device in that case by dissolution in acetone has a minimal effect on other polymers and metals in the structure.

The proposed process flow, with 2-step hard-baking for the sacrificial photoresist, successfully produced released test structures made of SiC, Al, and Ti. All of the structural layers survived the release process in which the sacrificial layer was dissolved in a photoresist remover ultrasonic bath. It is worth noting that photoresist remover 1165 (used for releasing the structures) has a negligible effect on a large range of materials, including metals, Si-based materials, and cured polymers such as Parylene C [16]. This shows the promising versatility of the proposed process in implementing a wide variety of surface micromachined devices. Table 1 lists typical choices of sacrificial layers, their corresponding etchants used in the release process, and possible structural layers that can be safely included in the process flow without being damaged during the release process. The proposed process flow is outlined in bold.

As seen in Table 1, the proposed sacrificial layer and its etching method accommodate a large range of structural layers within the suggested thermal budget. This process was validated by adapting it to the SiC surface micromachining technology developed by Nabki et al. in [10] which has a 180 °C max processing temperature. However, from an etching selectivity point of view, more structural layers can be supported as well. The usage of a wet release in the process flow results in a reduced etch time, and potential cost reduction. This is because the wet release requires 90 min, including the ultrasonic bath and critical point dryer (CPD) steps. In comparison, it is expected that the same released device areas and sacrificial gap size would require at least 10 h with dry-etching in an oxygen plasma. Moreover, oxygen plasma attacks cured polymers, thus ending the possibility of integrating them as structural layers, as listed in Table 1.

## 5. Conclusions

A hard-baked Shipley 1805 positive photoresist was used as a sacrificial layer for a low-temperature (i.e., sub-180 °C) surface micromachined process. The sacrificial layer was released in a photoresist remover in an ultrasonic bath. The proposed wet-release process requires much less time than the dry-release used for cured polymers, resulting in a significant potential reduction in the fabrication cost, and allows for cured polymers to be used as structural layers. The proposed process is expected to accommodate cured polymers such as Parylene and polyimides. It is also suitable for monolithic integration above CMOS and flexible substrates. The released test structures contained a combination of ceramic structural material (i.e., SiC) and conducting metals (Ti and Al). These three layers were unaffected by the proposed release process. 

## Figures and Tables

**Table 1:** Possible structural layers supported by different sacrificial layers, including that proposed in this work (**bold**).

Ref.	Sacrificial Layer	Sacrificial Layer Etchant	Possible Structural Layers
[9]	Poly-Si	TMAH, KOH	SiC, SiN
[5]	Poly-Si	TMAH	Oxide
[7,9]	Oxide	HF	SiC, SiN, Poly-Si, TiN
[13]	Oxide	3% HF, Pad etch	Ti, Al
[4,10,11]	Cured polymers	Oxygen plasma	Ti, Al, SiC, SiN
[14,15]	Photoresist + 115 °C hard-bake	Resist remover/Acetone + CPD	Ti, Al, Cured polymers
**This work**	**Photoresist + 175 °C hard-bake**	**Resist remover + hot ultrasonic bath + CPD**	**Ti, Al, SiC, Cured polymers**
